# Infection prevention and control across the continuum of COVID‐19 care: A qualitative study of patients', caregivers' and providers' experiences

**DOI:** 10.1111/hex.13558

**Published:** 2022-07-12

**Authors:** Marina B. Wasilewski, Zara Szigeti, Christine L. Sheppard, Jacqueline Minezes, Sander L. Hitzig, Amanda L. Mayo, Lawrence R. Robinson, Maria Lung, Robert Simpson

**Affiliations:** ^1^ St. John's Rehab Research Program Sunnybrook Research Institute Toronto Ontario Canada; ^2^ Department of Occupational Science and Occupational Therapy, Temerty Faculty of Medicine University of Toronto Toronto Ontario Canada; ^3^ Rehabilitation Sciences Institute, Faculty of Medicine University of Toronto Toronto Ontario Canada; ^4^ Musculoskeletal/STAR Rehab and Restorative Transitional Unit St. John's Rehab, Sunnybrook Health Sciences Centre Toronto Ontario Canada; ^5^ Division of Physical Medicine and Rehabilitation, Department of Medicine, Temerty Faculty of Medicine University of Toronto Toronto Ontario Canada

**Keywords:** continuum of care, COVID care quality, human connection, infection prevention and control, patient safety, rehabilitation

## Abstract

**Introduction:**

Healthcare facilities adopted restrictive visitor policies as a result of the COVID‐19 (COVID) pandemic. Though these measures were necessary to promote the safety of patients, families and healthcare providers, it led to isolation and loneliness amongst acute care inpatients that can undermine patient rehabilitation and recovery. The study objectives were to (1) explore how infection prevention and control (IP&C) measures impacted stakeholders' perceptions of care quality and interactions with others and (2) investigate how these experiences and perceptions varied across stakeholder groups and care settings.

**Methods:**

A qualitative descriptive study was conducted. Patients and their families from an inpatient COVID rehabilitation hospital and healthcare providers from an acute or rehabilitation COVID hospital were interviewed between August 2020 and February 2021.

**Results:**

A total of 10 patients, 5 family members and 12 healthcare providers were interviewed. Four major themes were identified: (1) IP&C measures challenged the psychosocial health of all stakeholders across care settings; (2): IP&C measures precipitated a need for greater relational care from HCPs; (3) infection prevention tenets perpetuated COVID‐related stigma that stakeholders experienced across care settings; and (4) technology was used to facilitate human connection when IP&C limited physical presence.

**Conclusion:**

IP&C measures challenged psychosocial health and maintenance of vital human connections. Loneliness and isolation were felt by all stakeholders due to physical distancing and COVID‐related stigma. Some isolation was mitigated by the relational care provided by HCPs and technological innovations used. The findings of the study underscore the need to balance safety with psychosocial well‐being across care settings and beyond the patient–provider dyad.

**Patient and Public Contribution:**

This study was informed by the Patient‐Oriented Research Agenda and developed through consultations with patients and family caregivers to identify priority areas for rehabilitation research. Priority areas identified that informed the current study were (1) the need to focus on the psychosocial aspects of recovery from illness and injury and (2) the importance of exploring patients' recovery experiences and needs across the continuum of care. The study protocol, ethics submission, analysis and manuscript preparation were all informed by healthcare providers with lived experience of working in COVID care settings.

## 1 INTRODUCTION

The COVID‐19 (COVID) pandemic has required a rapid and drastic response by healthcare systems worldwide, including major changes in how organizations and staff function to deliver patient care.^1^ The infection prevention and control (IP&C) measures used to enhance the safety of patients, caregivers and healthcare providers (HCPs) were unprecedentedly widespread and generated unique challenges for all stakeholders. One of the most prominent IP&C measures that was adopted worldwide involved strict ‘no visitor’ policies.^2^, ^3^ While IP&C measures like physical distancing, isolation, use of personal protective equipment (PPE) (e.g., masks, face shields, gowns) and visitation restrictions created important layers of protection, they undoubtedly resulted in negative consequences for patients such as anxiety, depression and loneliness.^4^, ^5^, ^6^

Visitation restrictions, in particular, proved to be especially challenging, as large‐scale disasters—such as the COVID pandemic—intensify stress and basic human needs to feel safe and connected.^7^ Balancing high‐quality care and human connection with the safety of patients, families and HCPs during a pandemic proved extremely challenging.^8^ HCPs were in an especially difficult position as they attempted to fulfil their own needs for human connection while simultaneously meeting those needs for their patients.^9^ This dichotomy between human connection and IP&C measures engendered feelings of mistrust, confusion and hurt amongst patients, families and HCPs alike.^10^ Although video conferencing technology was recognized as having the potential to reduce the risks associated with patient isolation,^2^ its success depended heavily on patient and families' existing technology access and digital literacy, as well as HCP availability to support implementation.^11^

While there is a growing body of evidence pertaining to the impact of IP&C measures during the early period of the pandemic, the majority of these studies have focused on single care settings (typically acute care and long‐term care), providing little insight into how IP&C measures impacted stakeholders' experiences both *within and across* care settings and how challenges precipitated by IP&C measures were addressed across settings. Further, most studies have focused on HCPs' perceptions of IP&C measures, revealing common themes around the need for resiliency in the face of resource and staff shortages, burnout and infection concerns to provide high‐quality patient care.^12^, ^13^, ^14^ However, very few studies have explored patient experiences with IP&C meaures,^6^ and none have included family caregivers' views. In turn, we investigated the COVID care pathway from the perspective of patients, caregivers and HCPs (‘stakeholders’). Specifically, our goals were to (1) explore how IP&C measures impacted stakeholders' perceptions of care quality and interactions with others and (2) investigate how these experiences and perceptions varied across stakeholder groups and care settings (i.e., acute care, inpatient rehabilitation and community).

## 2 METHODS

This paper draws on data from a study investigating the implementation and impact of COVID care within a hospital network comprised of an acute care and inpatient rehabilitation facility based in Toronto, Canada. We used a qualitative descriptive approach, which entails a concise and descriptively rich analysis that remains true to participants' own words. Thus, it produces a data‐near report that is representative of participants' views, making it meaningful for key stakeholders and relevant for justifying actionable change.^15^, ^16^

### 2.1 Participants

We recruited HCPs working in or supporting an acute or rehabilitation COVID unit by email using the hospitals' COVID unit listservs. We recruited patients from a database of people discharged from inpatient COVID rehabilitation between March and September 2020. We contacted patients via telephone or email. All patients were initially admitted to an acute care facility with a COVID diagnosis before being discharged to the inpatient rehabilitation hospital's COVID unit. When we contacted patients, we also asked for their caregiver's information (either telephone or email) to enable us to recruit them. Patients were eligible to participate if they were English speaking, cognitively able to provide consent and had been discharged in the past 6 months. Caregivers were eligible if they were a friend or family member supporting a patient who fulfilled these criteria and were themselves English speaking and cognitively able to provide informed consent.

### 2.2 Data collection

This study was approved by the Research Ethics Board at Sunnybrook Health Sciences Centre. Informed consent was obtained before data collection. One trained qualitative researcher (S. G.) conducted all interviews via telephone or Zoom between August 2020 and February 2021 (see Table 1 for an overview of the interview guide). One pilot interview was conducted with each stakeholder group, followed by discussion between the interviewer and the first author to ensure that the questions were clear and eliciting appropriate responses. These consultations indicated that no major modifications to the interview guide were required. Data were collected until saturation of ideas was reached. The interviewer and the research team were embedded within the inpatient rehabilitation hospital, and participants had no prior relationship with the interviewer and understood that the study goals were to explore stakeholder experiences with COVID care. Interviews ranged from 30 to 80 min, were audio‐recorded and transcribed verbatim. All identifying information was removed from the transcripts, and transcripts were uploaded to NVivo for organization and analysis. Sociodemographic information was collected from patients and family caregivers, and clinical characteristics of the patients were also collected. We also collected professional practice information from HCPs (e.g., profession, years of practice, practice setting).

**Table 1:** Semi‐structured interview questions for stakeholders

**Stakeholder group**	**Interview questions**
Patients	Could you tell me a little bit about your COVID‐19 care journey? Could you tell me what happened when you arrived at (acute care site)? What was it like receiving care at (acute care site) for COVID‐19? Overall, what went well during your stay at (acute care site) and what could have been improved? Can you tell me a little bit about your experience of preparing to leave (acute care site) to go rehab? How prepared did you feel for the transition to rehab? How did the transition go? What was it like receiving care at (rehab facility) for COVID‐19? Overall, what went well during your stay at (rehab facility) and what could have been improved? How did you feel about getting ready to go home? How did the discharge to home go? After returning to home, did you feel like you had the necessary information and resources to support your continued recovery from COVID‐19? Once you were home, what were your top concerns and needs?
Caregivers	Could you tell me a little bit about you and your loved one's COVID‐19 care journey? Could you tell me what happened when your loved one arrived at (acute care site)? What was it like to support your loved one while they were receiving care at (acute care site) for COVID‐19? Overall, what went well during your loved one's stay at (acute care site) and what could have been improved? Can you tell me a little bit about your experience as your loved one was preparing to leave (acute care site) to go rehab? How prepared did you feel for your loved one's transition to rehab? How did the transition go? What was it like to support your loved one while they were receiving care at (rehab facility) for COVID‐19? Overall, what went well during your loved one's stay at (rehab facility) and what could have been improved? How did you feel about your loved one getting ready to go home? How did the discharge to home go? After your loved one returned to home, did you feel like you had the necessary information and resources to support their continued recovery from COVID‐19? Once your loved one was home, what were your top concerns and needs?
HCPs	To start, could you tell me a little about your clinical role and work history? What were your thoughts and expectations when you first heard that a portion of (unit) was going to be converted into a COVID zone? What were your top concerns, questions, and needs when you learned that you would be working in the COVID zone? How do feel your needs/questions/concerns were addressed by managers, administrators and other senior leaders? o For managers/administrators/senior leaders: How did you address the needs/questions/concerns that staff had? What has the actual experience of working in the COVID zone with COVID patients been like? What are your thoughts on the extent and quality of care that was delivered to COVID patients? Reflecting on your experience with working in the COVID zone, what do you think the top successes were? Reflecting on your experience with working in the COVID zone, what do you think the top areas for improvement were?

Abbreviation: HCP, healthcare provider.

### 2.3 Data analysis

We used an inductive thematic approach following the steps outlined by Braun and Clarke,^17^ whereby data were deconstructed into isolated fragments, followed by reconstruction into overarching themes that describe the higher‐level messaging in the data. Two independent researchers (Z. S. and S. G.) completed the coding process and three additional researchers (C. S., R. S., M. B. W.) participated in the thematic analysis.

### 2.4 Rigour

Analytic rigour was enhanced by triangulating between multiple individuals throughout analysis, having regular team meetings and exercising reflexivity (discussing and journalling the study team's own biases and experiences that may influence data interpretation). We also adhered to the COREQ reporting guidelines (see Appendix SA).

## 3 RESULTS

In total, we interviewed 27 participants for this study, which included 10 patients and 5 caregivers (see Table 2 for patient and caregiver characteristics). Thirteen patients and caregivers were eligible for the study, but were either not interested (*n* = 9) or could not be reached (*n* = 4). We also recruited 12 HCPs. HCPs were occupational therapists (*n* = 3), patient care managers (*n* = 2), registered nurses (*n* = 2), medical department heads (*n* = 2), collaborative practice leaders (*n* = 2) and a pharmacist (*n* = 1). All HCPs (*n* = 12) reported graduate‐level education. With the introduction of a highly infectious and novel virus, governments and hospitals implemented measures that were aimed at prioritizing safety and minimizing the risk of viral transmission. These measures changed over time as an increased understanding of the virus emerged. Some of these measures included maintaining a distance of at least 6 feet from nonhousehold members (‘physical distancing’), avoiding crowding, wearing masks and restricted visitation policies in healthcare settings. Below, we describe three key themes that capture participants' experiences with receiving COVID care across the continuum of care and in the context of IP&C measures used.

**Table 2:** Demographic information of patient and caregiver participants (*n* = 15)

Characteristic (mean, SD)	Patient (*n* = 10)	Caregiver (*n* = 5)
Age in years	62.8 (17.9)	60.2 (4.3)
Length of stay in rehabilitation in days	12.4 (1.8)	n/a

Characteristic (*n*, %)	**Patient (*n*** = **10)**	**Caregiver (*n*** = **5)**
Positive COVID status upon admission to the inpatient rehabilitation hospital COVID unit	4 (40)	n/a
Sex		
Male	2 (20)	2 (40)
Female	7 (70)	3 (60)
Did not disclose	1 (10)	–
Birth country		
Canada	3 (30)	3 (60)
China	2 (20)	–
Guyana	1 (10)	–
Nigeria	1 (10)	1 (20)
Philippines	2 (20)	1 (20)
USA	1 (10)	–
Ethnicity		
Black	1 (10)	1 (20)
Chinese	2 (20)	–
Filipino	2 (20)	1 (20)
Indian	1 (10)	–
South Asian	1 (10)	–
White	3 (30)	3 (60)
Marital status		
Married	2 (20)	5 (100)
Widowed	4 (40)	–
Single	2 (20)	–
Common law	1 (10)	–
Did not disclose	1 (10)	–
Education		
Some high school	3 (30)	–
Completed college	3 (30)	2 (40)
Completed university	3 (30)	3 (60)
Graduate programme	1 (10)	–
Yearly income (Canadian dollar [CAD])		
10,000–19,999	2 (20)	–
20,000–29,999	2 (20)	–
30,000–39,999	2 (20)	1 (20)
40,000–49,999	–	1 (20)
50,000–59,999	–	–
60,000–69,999	1 (10)	2 (40)
Did not disclose	3 (30)	1 (20)

### 3.1 Theme 1: IP&C measures challenged the psychosocial health of all stakeholders across care settings

Participants' narratives highlighted that during the initial waves of the pandemic, health policies and guidelines were focused on restricting spatial movement and maintaining a safe distance between individuals for IP&C purposes. To spread individuals out in the acute and inpatient rehabilitation settings, patients had ‘their own rooms to minimize the risk of transmission’ (HCP02), rooms with windows that could not open, doors that were only opened for brief, distanced ‘check‐ins’ and no one to talk to in person. While physical separation was necessary to limit the potential spread of infection, all participants spoke to how this unintentionally resulted in social isolation and loneliness and created a pronounced need for human connection. Unsurprisingly, patients described feeling ‘lonely’, ‘depressed’, ‘isolated’ (PT05, PT11, PT15, PT18) and ‘sheltered’ (PT05) in both acute care and inpatient rehabilitation.

Outside visitors, including caregivers and loved ones, were also restricted from entering both the acute care and inpatient rehabilitation hospitals. This was described as ‘difficult for us and I'm sure for all the families and for the patients not to be able to have even one visitor’ (CG03). Restricted visitation by loved ones led to patients ‘wailing’ (HCP07) and being ‘visibly sad’ (HCP05). HCPs also expressed frustration at the physical separation that patients and families endured and stated that there were:

Ridiculous rules with visitation and not letting families come because they may unknowingly transmit the virus. But it didn't make sense. Especially if you're telling me if I have all the right PPE, I can go assess the patient. Why can't a family member, if they're provided with the right PPE, why can't they even go in and say, goodbye?. (HCP10)

This sense of isolation extended to HCPs as well. HCPs reported feeling lonely and socially isolated from their loved ones as a result of physical distancing guidelines. One HCP stated how she ‘really had to shelter ourselves from our parents’ and that it was ‘hard on us since we couldn't visit them’ (HCP01). HCPs spoke about their fears of unknowingly bringing home COVID and exposing their loved ones, highlighting the challenge of balancing safety (through physical distancing and limited contact) with maintaining important human connections. Others recognized how important it was to minimize social isolation, and described how they would be ‘miserable without [my family and husband]’ (HCP07) if they adhered to rigid physical distancing measures in their personal lives.

### 3.2 Theme 2: IP&C measures precipitated a need for greater relational care from HCPs

Visitor restrictions and distancing requirements made it such that HCPs had to play a substantial role in providing relational care to patients (e.g., maintaining their psychosocial well‐being, acting as proxy family, providing physical touch). The absence of family due to visitor restrictions meant that patients did not have someone readily available to talk to them to address their fears and anxieties. HCPs in both acute care and inpatient rehabilitation acknowledged this gap in emotional support, and out of recognition that patients were ‘afraid’ (HCP11, PT05, CG10), they offered reassurance and emotional support to their patients in times of distress. Patients described how they felt calmer, more comfortable and better cared for when their healthcare team verbally acknowledged their fears and trauma, and tailored their care strategies in response to these vulnerable emotional states.

While relational care needs were high in both acute care and inpatient rehabilitation, most patients felt that rehabilitation HCPs prioritized relational care in the face of IP&C rules. One patient highlighted how an inpatient rehabilitation HCP ‘came up to me and gave me a big hug. She had a hat and was gowned and a face covering and everything. But she still hugged me’ (PT05). Other patients described similar experiences, where they were ‘really emotional, and somebody came and reassured me… She was holding my hand, which I didn't think they were supposed to do […] but she told me it's okay… You can do it. So, I feel good, I feel better’ (PT11). Despite PPE creating challenges in terms of visual communication cues and physical distancing reducing physical touch, inpatient rehabilitation HCPs still ‘found a way’ to facilitate relational care for patients by supporting religious rituals (HCP08), treating patients with ‘kindness’ (PT17) and ‘niceness’ (PT20), and providing ‘personal touches’ such as helping patients style their hair (CG05).

Relational caring was also found in interactions where patients were able to discuss life outside of the hospital with HCPs—particularly in inpatient rehabilitation. When asked about why it was important for HCPs to discuss common interests with their patients, PT07 stated that it made her feel more ‘comfortable’ and that ‘the nurses actually wanted to interact with me. It didn't feel forced’. Caregivers similarly expressed appreciation for the relational care offered by HCPs—especially in their absence—noting that these actions were ‘meaningful to her [patient]. Helped her morale’ (CG05).

### 3.3 Theme 3: Infection prevention tenets perpetuated COVID‐related stigma that stakeholders experienced across care settings

Participants described their experiences of being stigmatized due to having had COVID, caring for someone who had COVID or working in a healthcare setting. Common to all participants, this stigma stemmed either explicitly from IP&C measures or implicitly from discriminatory infection prevention behaviours (e.g., distancing from someone who previously had COVID despite them no longer being infectious). Stigma was highly apparent in acute care settings, where IP&C measures were most strictly enforced due to heightened potential of infection amongst patients and risk to HCPs’ health. One patient described how, in acute care, due to physical distancing rules, ‘the doctor would just stand in the doorway. He didn't want to come near me […] it's just so upsetting, they were talking about me as though you don't know anything […] they think I don't understand, but I know you're gossiping about me’ (PT11). Many caregivers were pained to hear about their loved one's experience of stigma and perceived mistreatment. For example, one caregiver described how his mother was hospitalized due to COVID, and while nearing the end of her acute hospitalization, she ‘wasn't COVID positive anymore, so people [shouldn't] be afraid to touch her, and to come in to close communication with her, and those kinds of things. But they still were scared of her […] and if they came near her, she was treated like something that is dirty […] seeing that made me sick’ (CG07). Hearing of the stigma that their loved ones encountered often made caregivers feel guilty for ‘dumping’ (CG10) or ‘abandoning’ (CG07) their loved ones in the hospital to receive care.

For HCPs, their place of work generated a great deal of stigma that resulted in them feeling socially excluded in their personal lives. Both acute and inpatient rehabilitation HCPs explained that the idea of working in a hospital where COVID patients were admitted was extremely off‐putting to their social circles. There was an assumption that if you work in a COVID unit or are in contact with COVID patients, you likely have the virus, leading to ‘a lot of stigmatization of nurses specifically who worked in COVID units, and no one wanting to see them’ (HCP11). Some HCPs went as far as concealing their occupation status to prevent friends and family from ‘just freaking right out, and then who knows what type of stuff they'd say about me. That I'm sick or that they never want to see me’ (HCP09). This ultimately made HCPs feel ‘alone’ (HCP04) and ‘unwanted’ (HCP10). Some HCPs even described exclusionary encounters in their daily lives with strangers and acquaintances. For example, HCP10 shared her stigmatizing experience of trying to find a new home in the middle of the pandemic while working in a COVID unit:

We sold our house just before the pandemic and [were] trying to look for a house […] a whole bunch of people didn't want me entering their house if they knew I worked on a COVID unit. […] They were like, we don't want her to look at the house, let alone live in the house […] I felt like no one wanted me there.

Patients and caregivers also described a number of stigmatizing encounters within the community. Similar to HCPs, much of this stigma stemmed from people being afraid of getting infected with COVID and, in turn, showing discriminatory infection prevention behaviour. Patients explained that friends and family did not want to interact with them because they had previously tested positive for COVID—even if they were no longer infectious. Patients described a ‘strange’ (PT01) feeling where individuals in the community would ‘put up their masks as soon as they saw me coming’ (PT01). This ‘strange’ feeling was echoed by other patients who experienced stigma and discrimination. One patient stated that ‘no one in my condo would even look at me’ and that ‘no one has come up to my apartment since I've been home from the hospital’ (PT06), illustrating patients' feeling that people were distancing themselves because of the stigma of COVID. As a result, patients went to great lengths, such as ‘asking the hospital to write me a letter saying I'm not contagious’ (PT01), to address the stigma that they were facing in the community. Similarly, people distanced themselves from family caregivers of COVID patients, leading caregivers to be ‘careful who we told about caring for [patient], because they wouldn't want to see us […] even though it was more than the 14 days at that point’ (CG03).

### 3.4 Theme 4: Technology was used to facilitate human connection when IP&C limited physical presence

In the first wave of the pandemic (March–August 2020), local hospitals developed initiatives that equipped inpatients with tablets during their stay. These tablet initiatives allowed patients to remain in contact with their loved ones and were mostly utilized and enjoyed in inpatient rehabilitation. Communicating using the tablets helped patients to feel that they ‘had someone on the outside that I know was there for me. That helped the loneliness’ (PT18). Both patients and caregivers felt that tablet initiatives helped mitigate the negative repercussions of restricted visitation, including patient isolation. Many commented on how joy was brought to a patient after hearing the ‘familiar voices’ (CG07) of family and friends and that they would ‘perk up’ (CG04) and ‘light up’ (CG07) after these calls. Some patients mentioned that they were surprised to find that technology worked as an alternative to in‐person connection: ‘It wasn't what I expected, but I felt the same. I didn't feel disheartened because I was still able to talk to my family and Facetime them’ (PT07). Caregivers spoke of the important role that these calls played in meeting their loved ones' social and emotional needs and how this beneficially contributed to functional and physical recovery. For example, one caregiver described that their loved one felt ‘uplifted’ after talking on the phone with family, which motivated them to participate ‘in physio or things like that’ (CG04).

While frontline providers were working in environments that put them at a heightened risk to contract COVID, many of them relied on technology to remain connected with their own vulnerable loved ones, such as aging parents or young grandchildren. When discussing their own personal use of technology to remain connected with loved ones, some described it as ‘essential […] because otherwise, I would have never seen my parents’ (HCP07), and that using technology was ‘all we could do in our virtual world […] it wasn't worth the risk of seeing them in person, so we made it work over the phone’ (HCP03). The hospital organization also used technology to facilitate connection amongst its staff by sharing positive news over email and hosting informal ‘staff wellness programs where our psychiatric colleagues stepped up and started doing Zoom therapy sessions […] We just talked about how we were feeling’ (HCP12).

Although technology helped to overcome some COVID‐related challenges, it had its limitations. First, not all patients had equitable access to technology. This was particularly prevalent in the initial weeks of the pandemic when both acute care and inpatient rehabilitation did not yet have tablet initiatives. During these initial weeks, some patients explained that they ‘had to bring a cell phone from home’ (PT07) or because they ‘were never offered an iPad, I used the hospital phone in my room’ (PT01). Second, many COVID patients were older adults who were not comfortable using technology (e.g., tablets) and either had to quickly learn how to navigate these tools or request assistance from already busy HCPs who ‘couldn't always help me much’ (PT05). Finally, for many COVID patients, health status was an issue. In the acute care setting, some ‘families found the iPad stuff very difficult. Especially if patients weren't doing well medically. Some of them opted not to do those kinds of visits. It was just too hard on everyone’ (HCP10). In other cases, acute care patients who were severely deconditioned were ‘too weak to hold the iPad’ (PT14) and lacked the energy to carry out conversations with loved ones. One caregiver explained that as her loved one's health improved in inpatient rehabilitation, ‘she was able to call me on her own, herself. [In acute care], she was never able to do that. I had to go through the hospital, and sometimes that was an inconvenience on both of us’ (CG07).

## 4 DISCUSSION

Our study explored the experiences of patients, family caregivers and HCPs with COVID care across the continuum of care. Our findings highlight that the COVID pandemic created conditions that required all stakeholders to balance safety with the need for human connection. The four themes that we identified underscored that (a) social isolation and loneliness were experienced by all stakeholder groups due to IP&C measures; (b) HCPs had to provide patients with a great deal of relational care to make up for family absence; (c) some infection prevention strategies and behaviours were discriminatory and perpetuated stigmatization of participants; and (d) technology helped overcome the challenges of physical separation to facilitate human connection.

While the need for IP&C measures is undeniable, the path forward must ensure that safety is appropriately and rigorously balanced with relational care and maintenance of human connection. Foundationally, healthcare rests on the notion of ‘human beings caring for human beings’.^18^ Thus, it is unsurprising that human connection was revealed to be so vital for stakeholders involved in COVID care.^18^ The large majority of the existing literature focuses on patient and provider relationships and how ‘human connection’ with a care provider can make a difference to a patient in distress.^19^, ^20^ While our study affirms that the relational care provided by HCPs filled a substantial gap in emotional support due to visitor restrictions, it also underscores that in the context of widespread physical and social distancing, there is also a heightened need for human connection that extends beyond the patient–provider dyad.

First, HCPs require human connection with family and friends to maintain their ability to provide humanistic and compassionate care to their patients. While conceptions of the ‘therapeutic relationship’ have centred on HCPs' duty to unselfishly meet the needs of their patient,^21^ this overlooks HCPs' own needs for human connection and the equally important duty to care for themselves.^21^, ^22^ During a crisis like the COVID pandemic, it became apparent that connecting with others took on an added level of importance for meeting this duty to oneself. Without these connections, HCPs can find themselves dispirited and experiencing heightened compassion fatigue and burnout,^21^ which has deleterious consequences for patient safety and recovery.^23^ Resilience can mitigate the negative impact of HCPs' compassion fatigue on patient care quality during the COVID pandemic.^24^ Increasing human connection during the pandemic has been noted as a key strategy for sustaining a resilient healthcare workforce.^25^ While some resilience‐building initiatives during the pandemic have integrated elements of human connection,^25^ all have focused on fostering connections between HCPs and their colleagues or with patients and family caregivers. These initiatives have yet to acknowledge and leverage HCPs' relationships *outside* of the workplace and the important role that they can play in enhancing HCP resilience and functioning *in* the workplace.^26^ This is especially important, given that the existing literature points to HCP resiliency being a key tool for managing IP&C‐related challenges to ensure quality patient care,^12^, ^13^, ^14^ which was echoed by patient participants in our study who felt that care was positively impacted by HCPs adapting and ‘finding a way’ to overcome IP&C challenges (e.g., communication difficulties due to PPE; physical distancing rules preventing therapeutic touch).

Second, patients and family caregivers need to feel connected during physical separation. Patients in our study were lonely, isolated and depressed due to physical isolation. Our findings also reflect the perspective of family caregivers, who described a great deal of guilt for seemingly ‘abandoning’ their loved ones in the hospital—especially when they heard about their experiences of stigma. These findings are consistent with studies that have shown that restrictive visitor policies are associated with tangible deficits in care quality, patient experience and patient safety.^18^, ^27^ For instance, literature focused on palliative care during the COVID pandemic has highlighted that family presence at the end of life provides caregivers and patients with important human connection that fosters comfort, lessens fears and honours the dying patient's personhood.^28^, ^29^ Given that fear was a palpable part of all study participants' experiences and both caregivers and HCPs spoke of the importance of maintaining patients' humanity, our study suggests that the human connection derived from family presence must be prioritized throughout the recovery process and across the care continuum—not just at the end of life. Although mitigating COVID spread must continue to be prioritized, it is now apparent that restrictive visitation policies are detrimental to patient care. Our study took place before vaccines and rapid antigen tests were widely available and, thus, we now have greater resources at our disposal to safely maintain family presence.^2^

In circumstances where the physical presence of family cannot be achieved, technology can serve as a tool to help overcome isolation and loneliness. However, issues of equity and accessibility must be considered when implementing technological innovations in the future. Organization‐sponsored programmes—like the tablet initiatives discussed by participants in our study—are one way to ensure more equitable access across patients and families regardless of personal circumstances. Our findings echo the existing literature emphasizing that even with access, virtual visits can be challenging for older adults and ill patients without a dedicated facilitator,^30^ which can inadvertently add to the workloads of strained HCPs.^2^ Without dedicated funding, staffing support and implementation planning, it will be difficult for digital innovations to achieve the positive outcomes intended.

The added burden of stigma on the mental health of patients, caregivers and HCPs is substantial and warrants attention.^26^, ^31^ The existing literature suggests that this stigma creates a hostile and unsupportive environment for all,^31^ forcing them to live a life that is far from ‘ordinary’^32^—sentiment shared by participants who felt that they were being treated ‘strangely’. Mental health assessments and treatments have been recommended to be integrated into hospital‐ and community‐based COVID care for patients and caregivers,^26^ but our findings suggest that this should be expanded to also include HCPs who work in COVID care settings. Further, broad and multidisciplinary interventions are needed to raise community awareness and educate the public about COVID infectivity to help alleviate fears, dispel myths and combat discriminatory behaviour.^33^

### 4.1 Strengths and limitations

A notable strength of this study is the inclusion of patients, caregivers and HCPs and the exploration of their experiences across care settings (i.e., acute care, inpatient rehabilitation and community). We were successful in achieving robust sample sizes for patient and HCP stakeholder groups; however, family caregivers could have been better represented to further saturate caregiving‐specific ideas. Participants in our study were English‐speaking and had mid‐to‐high socioeconomic status (SES). Thus, our findings are limited in their transferability to linguistically diverse individuals and those from lower SES. The homogeneity of the sample may also explain the homogeneity of the results. Finally, our study represents a ‘snapshot’ of a specific period during the pandemic, and thus the perspectives of stakeholders may change or evolve as policies and procedures are modified based on emerging knowledge of COVID.

## 5 CONCLUSION

The safety measures needed to mitigate COVID spread created an environment that challenged psychosocial health and maintenance of vital human connections. Loneliness and isolation were felt by all stakeholders due to physical distancing and COVID‐related stigma. Some isolation was mitigated by the relational care provided by HCPs and technological innovations used. Our study points to the need to balance safety with humanity—both within and outside of the clinical setting. Multidisciplinary initiatives can mitigate the deleterious impacts of stigma on individuals' mental well‐being, and technology can be used for community outreach and to enable human connections.

## AUTHOR CONTRIBUTIONS

Marina B. Wasilewski was responsible for conceptualizing the study, funding acquisition, supervision, developing the methodology, formal analysis, interpretation of the data, drafting the manuscript and reviewing and editing the manuscript. Zara Szigeti was responsible for formal analysis, interpretation of the data, drafting the manuscript and reviewing and editing the manuscript. Christine L. Sheppard was responsible for developing the methodology, formal analysis, interpretation of the data and reviewing and editing the manuscript. Robert Simpson was responsible for conceptualizing the study, developing the methodology, formal analysis, interpretation of the data and reviewing and editing the manuscript. Jacqueline Minezes was responsible for conceptualizing the study, developing the methodology, interpretation of the data and reviewing and editing the manuscript. Sander L. Hitzig was responsible for conceptualizing the study, developing the methodology, interpretation of the data and reviewing and editing the manuscript. Amanda L. Mayo was responsible for conceptualizing the study, developing the methodology, interpretation of the data and reviewing and editing the manuscript. Lawrence R. Robinson was responsible for conceptualizing the study, developing the methodology, interpretation of the data and reviewing and editing the manuscript. Maria Lung was responsible for developing the methodology, interpretation of the data and reviewing and editing the manuscript.

## CONFLICT OF INTEREST

The authors declare no conflict of interest.

## Supporting information

Supporting information.

## Data Availability

The data that support the findings of this study are available on request from the corresponding author. The data are not publicly available due to privacy or ethical restrictions.
